# Pressure-Stabilized MnSb_2_ with Complex Incommensurate Magnetic Order

**DOI:** 10.1021/acs.jpcc.6c03519

**Published:** 2026-09-01

**Authors:** Mingyu Xu, Matt Boswell, Qing-Ping Ding, Cheng Peng, Aashish Sapkota, Qiang Zhang, Danielle Yahne, Sergey. L. Bud’ko, Yuji Furukawa, Paul. C. Canfield, Raquel A. Ribeiro, Weiwei Xie

**Affiliations:** † Department of Chemistry, 3078 Michigan State University, East Lansing, Michigan 48824, United States; ‡ Ames National Laboratory, 1177 Iowa State University, Ames, Iowa 50011, United States; § Department of Physics and Astronomy, Iowa State University, Ames, Iowa 50011, United States; ∥ Neutron Scattering Division, 564374 Oak Ridge National Laboratory, Oak Ridge, Tennessee 37831, United States

## Abstract

Marcasite-type compounds have been proposed as promising hosts of exotic magnetic quantum states, yet experimental realizations in stoichiometric, disorder-free systems remain limited. Here, we report the high-pressure stabilization and magnetic characterization of MnSb_2_, a marcasite-type compound that is thermodynamically metastable under ambient pressure. Single crystals were synthesized using a cubic multianvil press at 3.3 GPa and 490 °C for 24 h, and powder and single-crystal X-ray diffraction confirm the orthorhombic *Pnnm* structure. These crystals are stable at ambient pressure for a long time up to between 450 and 500 K. Heat-capacity measurements reveal phase transitions at approximately *T*_0_ ∼ 118 K and *T*_1_ ∼ 220 K. Neutron diffraction uncovers an unconventional magnetic state below *T*_1_ ∼ 220 K. Magnetic powder neutron diffraction refinements reveal possible multiple magnetic configurations that provide comparably acceptable fits to the experimental data. While most solutions are consistent with a spin-density-wave (SDW) description, helical models systematically yield inferior agreement factors. Across a broad range of models, the Mn ordered moment reaches a maximum value of approximately 2 μ_B_ and remains predominantly collinear, with minimal canting along the *c*-axis. At 200 K, the magnetic propagation vector is *q* = (0, 0.3975, 0.3783); upon cooling, the b component increases toward 0.5, reflecting a temperature-dependent evolution of the modulation. The need for modification of the magnetic model between high and low temperatures further highlights the complex and strongly temperature-dependent nature of the magnetic order in this system. These results establish MnSb_2_ as a pressure-stabilized marcasite magnet with a tunable, complex magnetic state and a compelling stoichiometric platform for exploring unconventional magnetic behavior, including potential altermagnetism.

## Graphical abstract

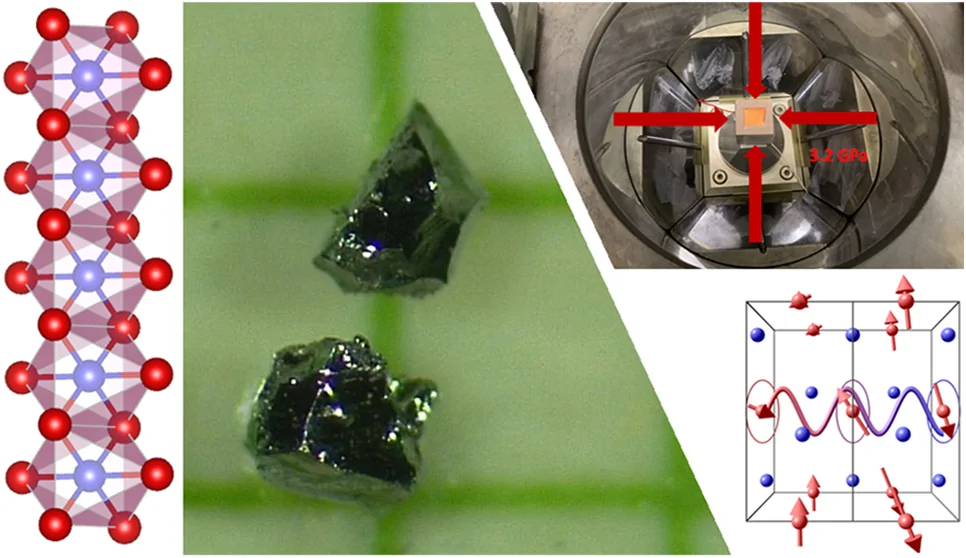

## Introduction

When magnetism intertwines with band topology in quantum materials, it can, in some cases, give rise to a recently identified class of magnetic systems characterized by collinear spin arrangements with zero net magnetization, exhibiting time-reversal-symmetry-breaking responses^1–8^ and spin-split electronic band structures.^9–16^ These properties, referred to as altermagnetism, combine zero-net-moment magnetic order with symmetry-allowed spin splitting relevant for spintronic applications and have motivated intense theoretical and experimental interest in identifying chemically clean material platforms that realize this magnetic phase.^8,17–31^ Marcasite-type compounds, in particular, have emerged as promising candidates due to their low crystal symmetry and symmetry-allowed spin splitting in antiferromagnetic states. FeSb_2_ is a narrow-gap, strongly correlated semiconductor^32–36^ that has been theoretically predicted to host a collinear altermagnetic state upon Co substitution or hole doping.^37^ Experimentally, however, FeSb_2_ exhibits no long-range magnetic order between 1.8 and 300 K. Stabilizing magnetic order in FeSb_2_ therefore requires deliberate tuning of its electronic structure. While chemical substitution and external pressure are widely used to modify band filling and bandwidth, substitution often introduces chemical disorder that complicates interpretation, and pressure-dependent magnetic measurements are experimentally nontrivial, especially for antiferromagnetism. This all motivates the search for chemically clean routes to stabilize magnetic order in the FeSb_2_ structure class.

A promising route to this goal is provided by MnSb_2_, a high-pressure marcasite-type compound that shares the same orthorhombic structure as FeSb_2_ but incorporates Mn with a larger, and often more robust, local magnetic moment and intrinsic hole doping^38^ (relative to FeSb_2_). Whereas MnSb_2_ does not form at ambient pressure, it can be synthesized under high-pressure conditions and quenched into a metastable form at ambient pressure.^39^ Across the marcasite family, CrSb_2_ exhibits robust antiferromagnetic order with a Néel temperature near 273 K,^40^ whereas FeSb_2_ remains nonmagnetic, suggesting that magnetic order emerges through band filling by hole doping. By analogy, MnSb_2_ represents a chemically ordered, stoichiometric platform in which unconventional states may be realized.

## Experimental Methods

### High-Pressure Synthesis

To improve phase purity and crystal quality compared with the one-step direct reaction reported^39^ at 6 GPa and 650 °C, MnSb_2_ was synthesized using a two-step method involving (i) the preparation of a homogeneous Mn–Sb precursor and (ii) a subsequent high-pressure, high-temperature reaction. In the first step, manganese metal (CZ-1–110) and antimony shot (99.999%) were combined in a 1:2 molar ratio and loaded into a 1.7 mL fritted alumina Canfield Crucible Set (CCS, LSP Industrial Ceramics, Inc.).^41^ The crucible was sealed in a silica ampule under an argon atmosphere (∼1/3 atm). Silica wool was packed around the CCS to stabilize the crucible during centrifugation and to contain any escaped liquid. The ampule was heated in a box furnace to 760 °C over 2 h, held at this temperature for 10 h, and then rapidly centrifuged to separate the molten and nonmolten phases. The solid residue retained on the frit disc appeared as a black solid, likely a minor oxide crust, while the solidified, decanted melt was collected, ground into a fine powder, and pressed into a pellet using a die press. This precursor step improves the compositional homogeneity of the starting material before the high-pressure reaction. In the second step, the pellet was loaded into a boron nitride (BN) crucible (7 mm length, 5.7 mm inner diameter), with any remaining void space filled with BN powder. The assembly was subjected to 3.3 GPa pressure at room temperature using a Rockland Research cubic multianvil press (∼20 mm anvil size) and then heated to 490 °C. This temperature was selected as the maximum before the Mn–Sb mix begins to melt, determined using the sample current (power) as a function of temperature curve, as shown in the Supporting Information. Maintaining the reaction below the melting temperature suppresses incongruent melting and the associated formation of Mn_1.1_Sb. After 24 h, the sample was quenched to room temperature before slowly releasing the pressure. This modified protocol yields a high-quality MnSb_2_ product, with more than half of the material consisting of submillimeter-sized single crystals. PXRD detects only approximately 1 wt % elemental Sb, while NMR indicates less than 1% Mn_1.1_Sb

### Single Crystal X-ray Diffraction

To determine the crystal structure of the obtained single crystal, the sample with dimensions 0.218 × 0.158 × 0.138 mm^3^ was picked up, mounted on a nylon loop with Paratone oil, and measured using an XtalLAB Synergy, Dualflex, Hypix single crystal X-ray diffractometer with an Oxford Cryosystems 800 low-temperature device. Data were collected using ω scans with Mo Kα radiation (λ = 0.71073 Å). The total number of runs and images was based on the strategy calculation from the program CrysAlisPro 1.171.43.92a (Rigaku OD, 2023). Data reduction was performed with correction for Lorentz polarization. The integration of the data using an orthorhombic unit cell yielded a total of 3540 reflections to a maximum θ angle of 40.13° (0.55 Å resolution), of which 478 were independent (average redundancy 7.406, completeness = 98.8%, *R*_int_ = 5.61%). A numerical absorption correction was applied based on Gaussian integration over a multifaceted crystal model.^42^ Empirical absorption correction used spherical harmonics, implemented in the SCALE3 ABSPACK scaling algorithm.^43^ The structure was solved and refined using the SHELXTL Software Package.^44,45^

### Powder X-ray Diffraction

To examine the phase information, the powder X-ray diffraction (PXRD) analysis was performed subsequent to the synthesis process. The crystals were ground using an agate mortar and pestle to achieve a homogeneous powder. This powdered sample was then uniformly distributed on a single crystalline silicon sample holder, designed for zero background measurements, with a minimal application of vacuum grease to secure the powder in place. The PXRD data acquisition spanned a 2θ range from 15° to 80°, utilizing incremental steps of 0.01° and a fixed dwell time of 3 s per step. These measurements were conducted using a Rigaku MiniFlex II powder diffractometer, employing Bragg–Brentano geometry coupled with Cu Kα radiation (λ = 1.5406 Å). The refinement of the powder X-ray data was executed using the GSAS-II software suite.^46^

### Physical Properties Measurements

Temperature-, magnetic-field-dependent DC magnetization data and resistance measurements were collected using Quantum Design (QD), Magnetic Property Measurement Systems (MPMS and MPMS3), and Physical Property Measurement Systems (PPMS). During the measurements of single crystals, the field is along a random direction of the crystals due to the poorly defined facets and the small size of the crystal. The samples are placed between two collapsed plastic straws, with the third uncollapsed straw providing support as a sheath on the outside or a quartz sample holder. Samples were fixed on the straw or quartz sample holder with GE-7031-varnish. AC electrical resistance measurements were performed in a standard four-contact geometry using the ACT option of the PPMS, with a 3-mA current and a frequency of 17 Hz. 50 μm diameter Pt wires were bonded to the samples with silver paint (DuPont 4929N) with contact resistance values of about 2–3 Ω. Temperature-dependent specific heat measurements on the MnSb_2_ in a mass of about 6 mg polycrystalline sample were carried out using a Quantum Design, Physical Property Measurement System (PPMS DynaCool).

### Neutron Powder Diffraction

To determine whether magnetic ordering exists, neutron powder diffraction (NPD) measurements were performed using a time-of-flight powder diffractometer, POWGEN, at the Spallation Neutron Source at Oak Ridge National Laboratory. Approximately 2.6 g of samples were prepared after grinding several single crystals to a fine powder and passing them through a 45 μm mesh sieve. Diffraction patterns were collected at 308 K for 2 h 36 min using a neutron beam with a center wavelength of 1.5. Rietveld refinements using GSAS-II software^46^ and Fullprof suite^47^ were performed to determine the crystal and magnetic structures, respectively. Determination of the magnetic structure was performed on HB-2A POWDER beamline at the High-Flux Isotope Reactor (HFIR) located at Oak Ridge National Lab (ORNL). Roughly 1 g of MnSb_2_ was loaded into an aluminum can backfilled with He. Diffraction patterns were measured with a vertically focused Ge monochromator to select the 2.41 Å wavelength while a collimation of open–open-12 was used. Magnetic structure analysis was performed with SARAh Representation Analysis and SARAh Refine and refined with the Fullprof suite software.^48^

### Nuclear Magnetic Resonance

Nuclear magnetic resonance (NMR) measurements of ^55^ Mn (nuclear spin *I* = 5/2, gyromagnetic ratio γ_N_/2π = 10.50 MHz/T), ^121^Sb (*I* = 5/2, γ_N_/2π = 10.189 MHz/T), and ^123^Sb (*I* = 7/2, γ_N_/2π = 5.517 MHz/T) nuclei were conducted using a laboratory-built phase-coherent spin–echo pulse spectrometer on polycrystalline powder samples. The NMR spectrum was obtained under magnetic fields by sweeping the magnetic field at a fixed NMR frequency of 67 MHz, while the zero magnetic field NMR spectrum was measured by plotting spin–echo intensity as a function of NMR frequency.

### DFT Calculation

Density Functional Theory (DFT) calculations were carried out using version 7.3.1 of the Quantum ESPRESSO package to evaluate the total energies of MnSb_2_ in nonmagnetic, ferromagnetic, and antiferromagnetic configurations.^49^ The calculations employed ultrasoft pseudopotentials and the Perdew–Burke–Ernzerhof (PBE) exchange-correlation functional within the generalized gradient approximation (GGA).^50–52^ A plane-wave kinetic energy cutoff of 300 Ry was used, with a charge density cutoff set to 3600 Ry (12 times the wave function cutoff). Brillouin zone integration was performed using a 7 × 7 × 13 Monkhorst–Pack *k*-point grid.^53^ Total energy convergence was ensured with a threshold of less than 1 meV per atom. The electronic self-consistency was achieved using the Davidson diagonalization algorithm, with a convergence criterion of 10^–9^ Ry.^54^ High-symmetrical *k*-point paths for band structure calculations were generated using the Spglib library.^55,56^

## Results and Discussion

MnSb_2_ was synthesized at 3.3 GPa pressure and 490 °C using a Rockland Research cubic multianvil press. After maintaining this temperature for 24 h, a high-quality MnSb_2_ product, with more than half of the final material consisting of submillimeter-sized MnSb_2_ single crystals that can be easily separated mechanically from the surrounding MnSb_2_ polycrystalline matrix, was obtained. (See the Experimental Methods section for further details.) The single-crystal X-ray diffraction (SCXRD) analysis of MnSb_2_, summarized in Tables S1 and S2, illustrates the crystal structure of MnSb_2_ shown in the right inset of Figure 1, which adopts the orthorhombic *Pnnm* space group and features edge-sharing MnSb_6_ octahedra. The unit cell contains two crystallographically distinct atomic sites: Mn atoms occupy the 2*a* Wyckoff position, while Sb atoms reside at the 4*g* positions. Vacancies and site mixing were considered during the refinement, but no structural disorder or clear vacancies were detected.

**Figure 1:**
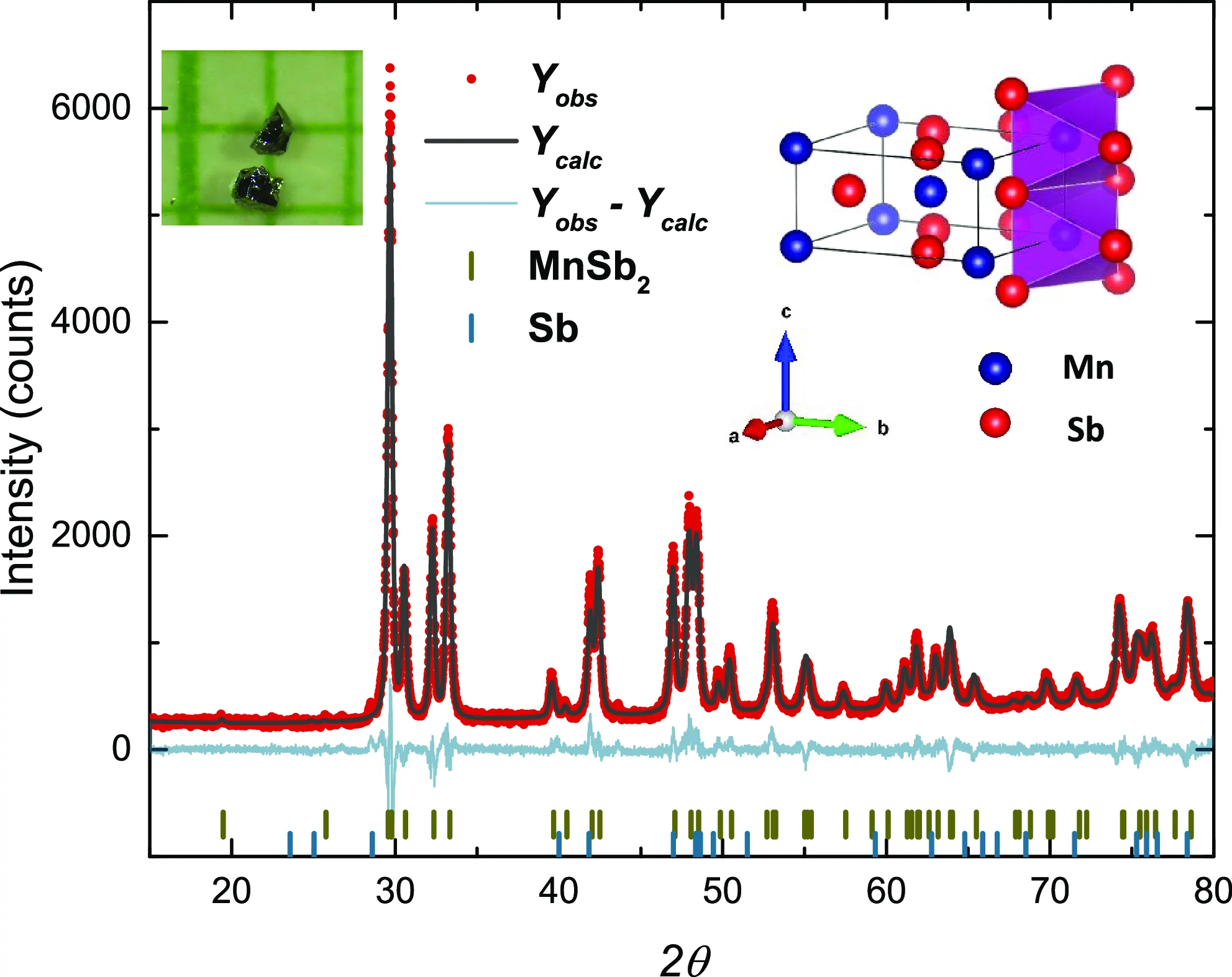
Phase identification and structural characterization of MnSb_2_. Powder X-ray diffraction pattern of MnSb_2_ with Rietveld refinement. Red circles indicate experimental data, the black line the calculated pattern, and the blue line the difference curve. The main phase is indexed to MnSb_2_, with a minor impurity phase identified as elemental Sb (about 1% by weight). *Right inset*: crystal structure of MnSb_2_. *Left inset*: optical image of representative single crystals; grid spacing is 1 mm.

To assess the phase purity of samples synthesized under high-pressure conditions, powder X-ray diffraction (PXRD) measurements were performed using a bulk sample with single crystals in the polycrystalline matrix, as shown in Figure 1. The experimental data (red circles) were analyzed by Rietveld refinement (black line) using the GSAS-II software package,^57^ and the difference between the observed and calculated patterns is shown by the blue line. The minor phase of elemental Sb is observed. The PXRD results are consistent with single-crystal X-ray diffraction measurements and with the previous report.^39^ The left inset of Figure 1 shows representative as-grown crystals, which display metallic luster and well-defined faceted morphologies, consistent with high crystallinity.

To determine the thermal stability range of MnSb_2_ at ambient pressure, temperature-dependent magnetization measurements were performed under both field-warming (FW) and field-cooling (FC) conditions, as shown in Figure 2a. Below 450 K, the magnetization curves exhibit no significant difference between the FW and FC protocols, indicating thermal and magnetic stability within this temperature range. However, above 450 K, a pronounced increase in magnetic moment is observed, suggesting the onset of decomposition and the formation of Mn_1.1_Sb, a known ferromagnetic impurity phase. This observation is corroborated by powder X-ray diffraction data collected before and after the magnetization measurements. As shown in Figure 2b, additional reflections corresponding to Mn_1.1_Sb emerge, and the intensity of the Sb peak increases significantly after heating above 500 K, confirming the partial decomposition of MnSb_2_. These results indicate that while MnSb_2_ is metastable at ambient pressure, it remains structurally and magnetically stable at temperatures up to ∼450 K, enabling long-term low-temperature studies without phase transformation. Moreover, within the temperature range of 1.8 to 300 K, magnetization measurements (see Figure 3) show no signatures of ferromagnetic or other phase transitions in MnSb_2_. The field-dependent magnetization displays predominant paramagnetic behavior with minor ferromagnetic contributions attributable to trace Mn_1.1_Sb impurities. Collectively, these results confirm that MnSb_2_ is suitable for extended investigations at low temperatures under ambient conditions, without undergoing structural or magnetic phase transitions.

**Figure 2:**
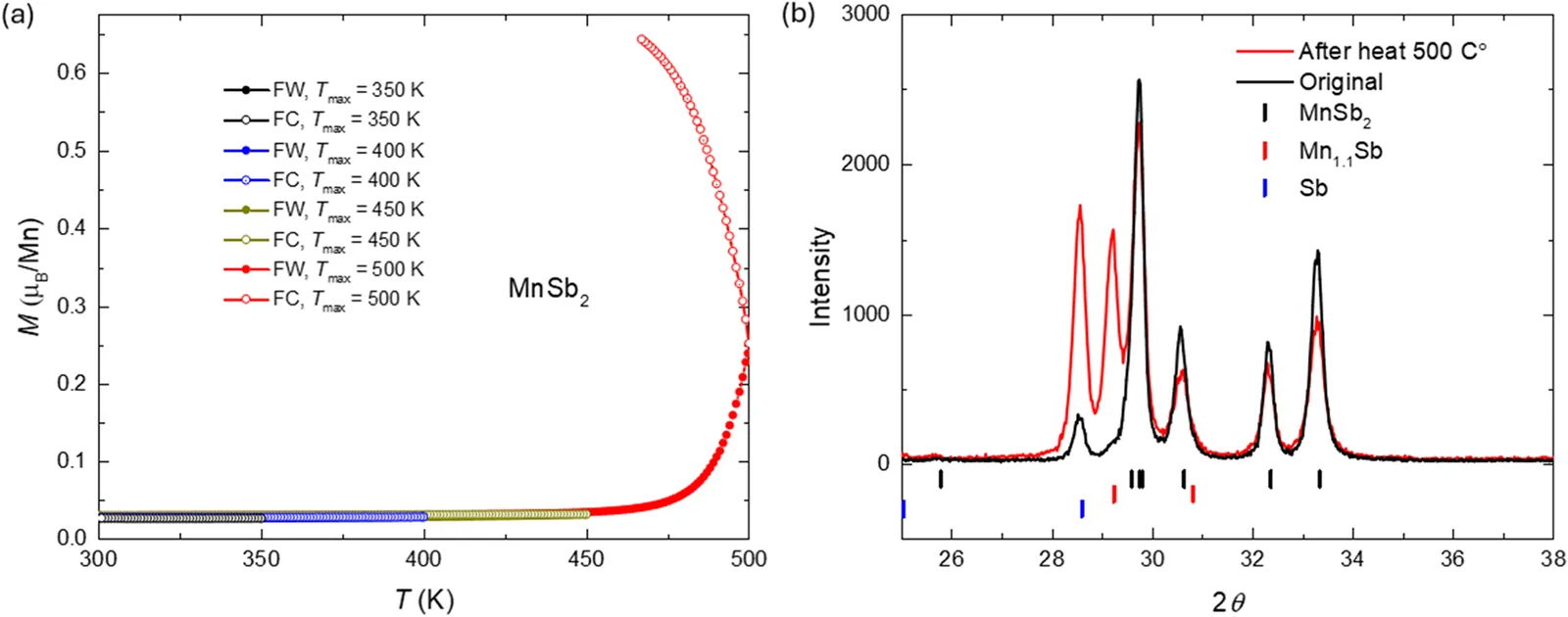
(a) Temperature-dependent magnetization of MnSb_2_ measured under field-cooling (FC) and field-warming (FW) across different temperature ranges: 300–350 K (black), 300–400 K (blue), 300–450 K (yellow), and 300–500 K (red). A significant increase in magnetization above 450 K indicates the onset of decomposition. (b) Powder X-ray diffraction patterns collected before and after the magnetization measurements are shown in (a). The emergence of Mn_1.1_Sb reflections and an increase in Sb peak intensity after heating above 500 K confirm the thermal decomposition of MnSb_2_.

**Figure 3:**
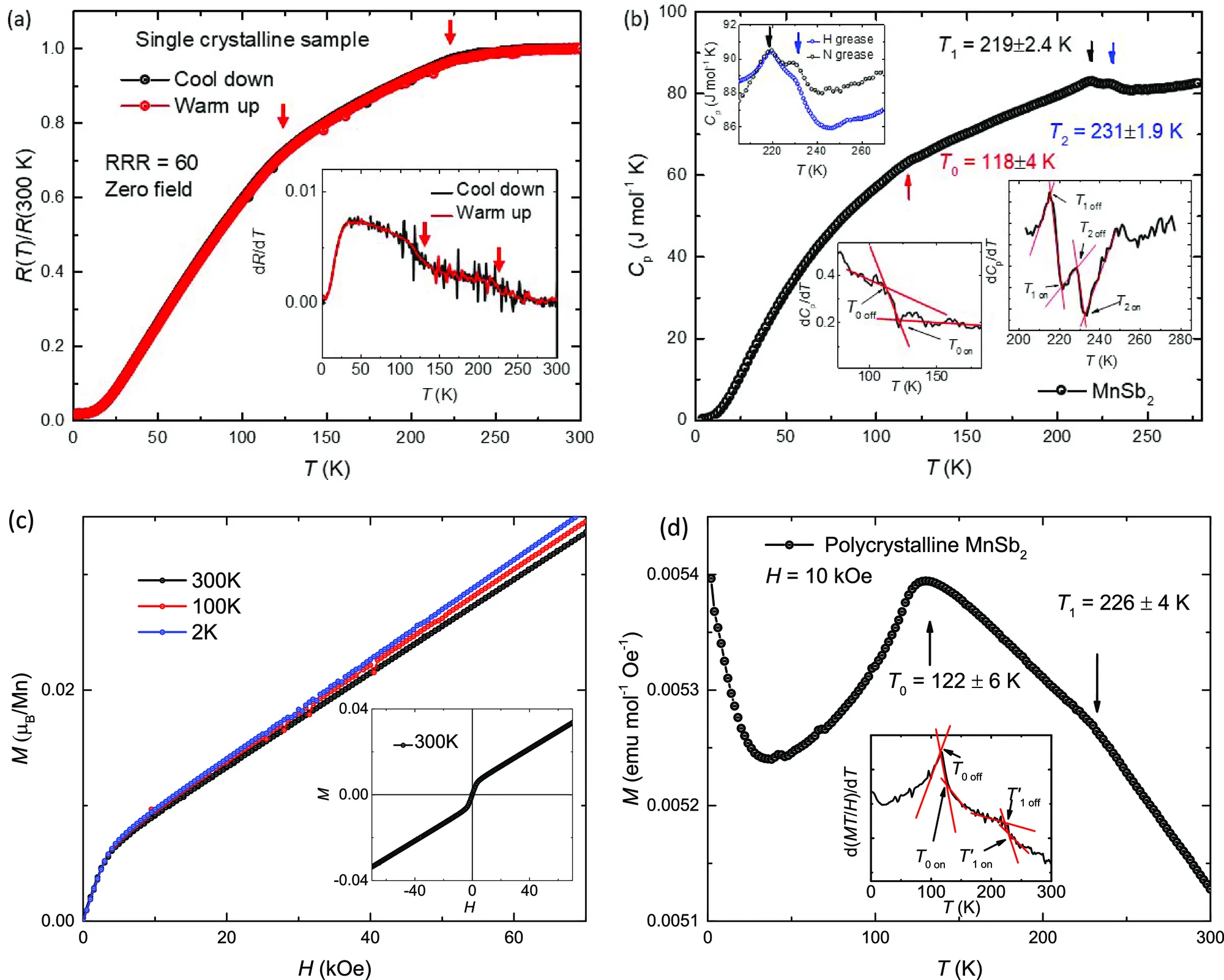
Electrical transport, specific heat, and magnetization of MnSb_2_. (a) Electrical resistance of single-crystalline MnSb_2_ measured upon cooling (black) and warming (red) between 300 and 1.8 K, showing negligible thermal hysteresis. *Inset*: Temperature derivative d*R*/d*T* with two anomalies. (b) Temperature-dependent specific heat showing three anomalies at *T*_0_, *T*_1,_ and *T*_2_, defined by the midpoint between onset and offset temperatures, shown in the right insets. Data from 200 to 280 K are obtained by point-by-point background subtraction of N-grease measurements. The temperature range is from 4K −280 K. The left inset shows a comparison of H-grease and N-grease measurements. *T*_1,_ and *T*_2_ anomalies are clearly shown in both measurements. (c) Field-dependent magnetization (*M–H*) of polycrystalline MnSb_2_ measured at selected temperatures. Inset: representative *M–H* curves at 300 K. (d) Temperature-dependent magnetization (*M–T*) of polycrystalline MnSb_2_ measured under applied magnetic fields. *Inset*: temperature derivative of M*T, showing two anomalies between 1.8 and 300 K. The black arrow indicates the anomalies shown in the measurement and the criteria of anomalies’ temperature and width.

To probe the magnetic and electronic properties of MnSb_2_, temperature-dependent electrical resistance measurements were performed on single-crystalline samples upon cooling from 300 to 1.8 K and during subsequent warming, as shown in Figure 3a. The resistance curves from the cooling and warming cycles overlap closely, indicating negligible thermal hysteresis. The residual resistance ratio (RRR), defined as R­(300 K)/R­(1.8K), is approximately 60. The inset of Figure 3a displays the temperature derivative of the resistance (d*R*/d*T*). There are two clear breaks in slope visible in the raw data and two broad steps in d*R*/d*T* around 118 and 220 K, as indicated with the arrows in Figure 3a and the inset. Complementary thermodynamic information is provided by temperature-dependent specific heat measurements on polycrystalline MnSb_2_ performed at zero magnetic field. As shown in Figure 3b, three reproducible anomalies are observed at approximately 118 K (*T*_0_), 219 K (*T*_1_), and 231 K (*T*_2_). These features are independently confirmed using an alternative measurement protocol over a different temperature range. Notably, the application of a magnetic field of 90 kOe produces negligible changes in specific heat (Figure S3a), demonstrating that the transition is largely insensitive to external magnetic fields. Integration of the excess specific heat yields a total magnetic entropy change of approximately *R* ln 6 (Figure S3b) using the reference of FeSb_2_ specific heat measurement, which is close to the Mn high-spin state. Curiously, the weak field dependence of the upper specific heat feature might suggest an antiferromagnetic transition that couples only weakly to uniform magnetic fields. To assess the uniform magnetic response of MnSb_2_ and the presence of any net magnetic moment, magnetic susceptibility and field-dependent magnetization measurements were performed. Figure 3c shows the field-dependent magnetization of polycrystalline MnSb_2_ measured up to 70 kOe at selected temperatures. The magnetization exhibits only weak temperature dependence remains unsaturated over the entire field range, and displays a very small net moment. A weak ferromagnetic contribution is observed and attributed to trace amounts (<1%) of the Mn_1.1_Sb impurity, which is below the detection limit of X-ray diffraction but was detected by NMR measurements (Figure S5). The inset shows representative *M*(*H*) curves measured at 300 K in five quadrants, confirming the absence of intrinsic ferromagnetic hysteresis. Figure 3d presents the temperature-dependent magnetization of polycrystalline MnSb_2_ measured under a 10 kOe applied field. There are two anomalies between 2 and 300 K, *T*_0_ = 122 K and *T*_1_ = 226 K, indicated by the black arrows. This observation is consistent with electric transport and the heat-capacity measurements. The heat-capacity data clearly resolve two closely spaced anomalies at *T*_1_ ∼ 219 K K and *T*_1_ ∼ 231 K. In contrast, the corresponding features in magnetization and resistivity are weaker and broader, and their widths are comparable to the separation between *T*_1_ and *T*_2_. Consequently, these measurements show only a single broad upper-temperature feature and cannot reliably distinguish the two transitions. The microscopic origin of *T*_2_ remains unresolved and may involve short-range magnetic correlations or a subtle structural response. The inset displays the temperature derivative of M*T, which shows two anomalies. This behavior is consistent with an amplitude-modulated antiferromagnetic ground state and highlights the limited sensitivity of uniform magnetization measurements to this type of magnetic order as well as a relatively large contribution to *M*(*T*) from a ferromagnetic impurity. To elucidate the microscopic origin of this magnetic transition, we next turn to neutron scattering measurements, which directly probe the magnetic ordering wave vector and spatial distribution of the ordered moments.

Powder neutron diffraction measurements on MnSb_2_ were carried out between 308 and 4 K (Figure 4). Upon cooling below ∼200 K, well below the ∼220 K upper magnetic transitions, additional Bragg reflections (shown in the red arrows) of magnetic origin emerge (Figure 4a). Similar magnetic peaks are found at 150 K and new peaks (shown in the black arrows) at 100 and 4 K, temperatures below the lower magnetic transition. Refinements of diffraction patterns collected above (Figures 4b) and below (Figure 4c) the show that orthorhombic MnSb_2_ develops a complex magnetic ground state that evolves with temperature.

**Figure 4:**
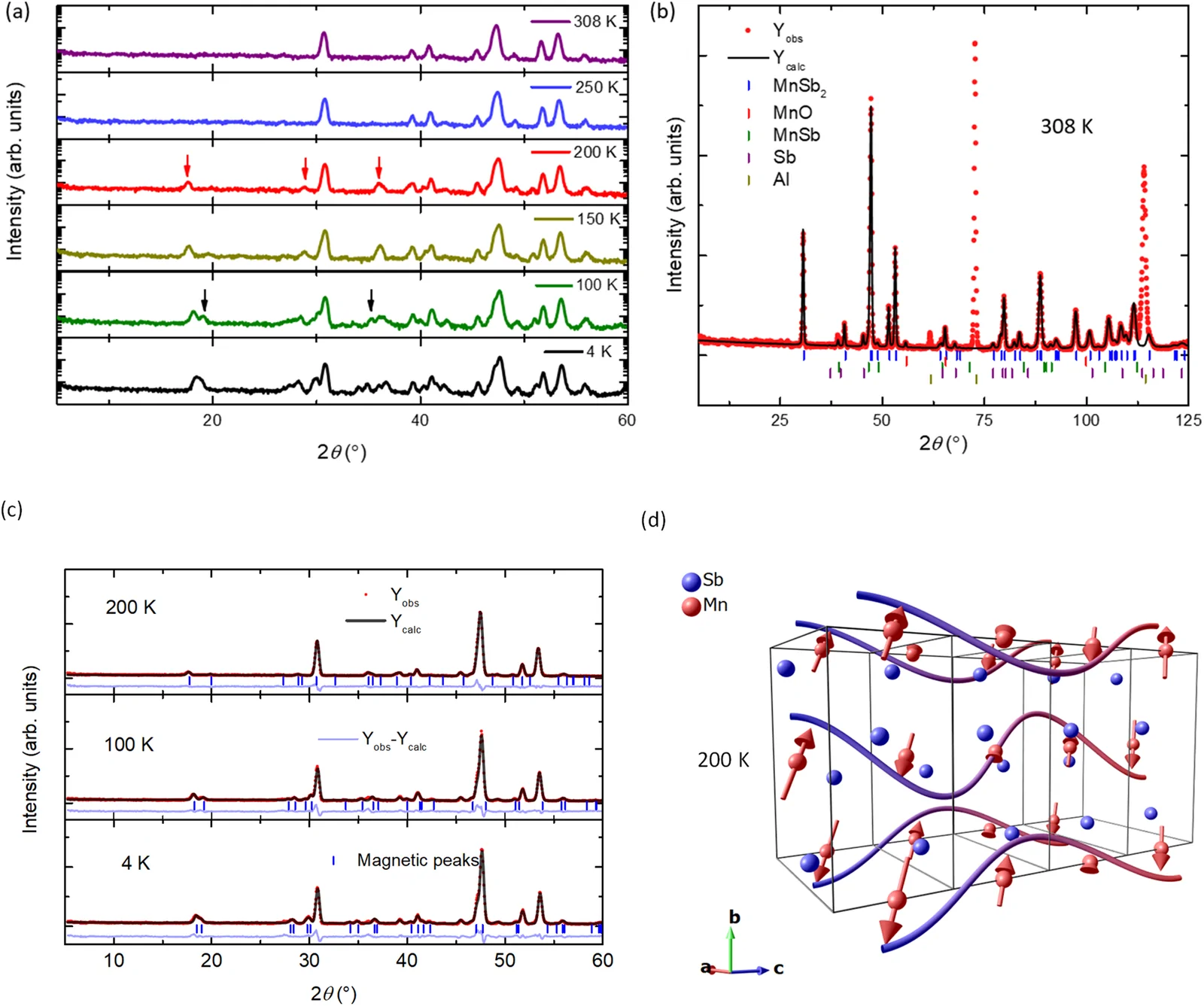
Neutron diffraction and magnetic structure of MnSb_2_. (a) Powder neutron diffraction patterns collected at 308, 250, 200, 150, 100, and 4 K on a logarithmic intensity scale. Peaks persisting at 308 K primarily correspond to nuclear reflections. Red arrows indicate the high-temperature magnetic reflections appearing below approximately 200 K, while black arrows indicate additional low-temperature magnetic reflections appearing below approximately 118 K. (b) Rietveld refinement at 308 K. The colored tick marks identify the nuclear reflections from MnSb2 and minor MnO, MnSb, and Sb phases; the Al reflections originate from the sample can. (c) Rietveld refinements at 200, 100, and 4 K. The blue vertical tick marks indicate the magnetic Bragg reflections. (d) Schematic representation of the magnetic structure of MnSb_2_ at 200 K.

At 200 K the propagation vector *q* is best described as (0, 0.3979 (4), 0.3776 (1)) while lowering the temperature to 4 K changes the propagation vector to (0, 0.4784 (3), 0.3752 (1)). The largest change occurs in the *b*-axis component, which continuously approaches the commensurate value of 0.5, whereas the *c*-axis component changes only slightly. This feature is reminiscent of Co_3_V_2_O_8_, which also has a continuous evolution of the propagation vectors and specific temperatures where it locks into a commensurate structure.^58^ Representational analysis performed using SARAh identified two irreducible representations, each containing three basis vectors,^59^ that together provide the best description of the magnetic reflections, as shown in Table 1. At 200 K, three combinations of mixing coefficients lead to nearly equivalent fits to the powder neutron data, as listed in Table S8*a*. One of these models produces a sinusoidally modulated spin-density-wave structure, shown in Figure 4d. where the *a*-component of irreducible representation 1 is mixed with the *b* and *c*-components of irreducible representation 2. A comparably good fit is obtained when the phase of the *a*-component is changed, resulting in a helical structure rotating about the *a*-axis. A third nearly equivalent model uses only irreducible representation 2, with *b* and *c*-components forming a sinusoidally modulated structure similar to the structure observed in Figure 4d. In all three models, the maximum refined magnetic moment is approximately 2.3 μ_B_ Because these models provide nearly indistinguishable fits to the powder data, the magnetic structure at 200 K cannot be determined uniquely. We therefore present the sinusoidal spin-density-wave model in Figure 4d as a representative description of the magnetic order, rather than a definitive structure assignment, with its plausibility additionally motivated by the metallic character of MnSb_2_.

**Table 1:** Symmetry-allowed irreducible representations and basis vectors at 200 K for the Pnnm space group with k-vectors (0 0.398 0.377). The ζ denotes the component as exp­(−2πik • t).

IR	BV	Mn (0.5 0.5 0)	Mn (0 0 0.5)
Γ_1_	ψ_1_	(1 0 0)	(ζ 0 0)
	ψ_2_	(0 1 0)	(0 −ζ 0)
	ψ_3_	(0 0 1)	(0 0 −ζ)
Γ_2_	ψ_4_	(1 0 0)	(−ζ 0 0)
	ψ_5_	(0 1 0)	(0 ζ 0)
	ψ_6_	(0 0 1)	(0 0 ζ)

As the temperature is lowered, the magnetic structure becomes more complex. At 4 K, additional basis vectors are required to reproduce the magnetic diffraction pattern. However, as at 200 K, different combinations of basis vectors and mixing coefficients can yield fits of comparable quality, and this ambiguity is even more pronounced at 100 and 4 K as shown in Table S8b. Although the exact low-temperature magnetic structure therefore cannot be uniquely determined from powder diffraction alone, the refinements generally favor helical models over purely sinusoidal ones. The best-fitting models typically involve a mixture of irreducible representations and yield maximum magnetic moments of approximately 2.7 μ_B_, with some variation depending on the specific basis vectors and mixing coefficients employed. In addition, several low-temperature peaks cannot be reproduced by the present model. These reflections may arise from a magnetic impurity phase or from a more complex magnetic structure, such as a multi-q state. A temperature-driven evolution from sinusoidal to helical magnetic order is not unprecedented and has, for example, been observed in the frustrated magnet Gd_2_PdSi_3_ and other frustrated magnet systems.60 Further neutron-scattering measurements, particularly polarized-neutron diffraction and ideally single-crystal measurements, would be required to better constrain the magnetic structure and distinguish among the competing models. A systematic comparison of the different basis-vector combinations and their corresponding refinement statistics is provided in the Supporting Information.

Within the limitations of powder neutron diffraction, the refined structure at 200 K can be narrowed down to a few structures but is best described as predominantly sinusoidal. However, an elliptical or helical structure cannot be excluded as a possibility. Therefore, we avoid assigning MnSb_2_ to an altermagnetic class based on the present data. Further single-crystal neutron diffraction is required to test whether MnSb_2_ exhibits symmetry-protected spin splitting or other features associated with altermagnetism.^60^

To evaluate the magnetic ground state of MnSb_2_, first-principles calculations were performed using the experimentally determined single-crystal structure, considering nonmagnetic (NM), ferromagnetic (FM), and a simple collinear antiferromagnetic (AFM) configurations with antiparallel Mn moments in the primitive cell. Although neutron diffraction reveals a considerably more complex magnetic structure, calculations with a simple collinear AFM configuration provide a useful reference for evaluating the magnetic instability of the electronic structure. In addition, the limited experimental constraints on the full magnetic structure from unpolarized neutron diffraction and the computational cost of large magnetic supercells make such simplified calculations a practical first step. The calculated total energies (Table S3) show that the AFM state is the most stable, lying 80.79 meV below the NM configuration, while the FM state is also energetically favored (69.68 meV below NM), indicating close energetic competition among magnetic states. In the AFM configuration, the calculated local magnetic moment on Mn is 2.81 μ_B_. The calculated Mn moment of 2.81 μ_B_ is reasonably close to the maximum ordered moment of approximately 2.7 μ_B_ obtained from neutron diffraction refinement at 4 K. The small difference likely reflects the simplified equal-moment collinear AFM configuration adopted in the DFT calculations compared with the experimentally observed incommensurate, amplitude-modulated magnetic structure. Because the experimental propagation vector is incommensurate and varies continuously with temperature, explicitly incorporating the experimentally determined magnetic structure into the calculations would require large commensurate supercell approximants and substantially greater computational resources. The collinear AFM model is therefore employed here as a simplified representation to examine the energetic stabilization of magnetism and its influence on the electronic structure near the Fermi level. The calculated electronic band structures are shown in Figure 5. In the NM state (Figure 5a), a flat band appears near the Fermi level, producing a pronounced peak in the density of states and signaling electronic instability. Although MnSb_2_ shares the same nominal valence electron count as FeSb_2_, the partial occupation of states at the Fermi level confirms metallic behavior rather than the insulating ground state of FeSb_2_. Upon inclusion of spin polarization in the AFM state (Figure 5b), the flat band shifts below the Fermi level, and the density of states at the Fermi level is strongly reduced, leading to the formation of a pseudogap and a more stable electronic structure. MnSb_2_ is a stoichiometric and chemically ordered compound with antiferromagnetic order and a very small net magnetization. The DFT calculations favor a compensated collinear AFM state and reveal a pronounced spin-polarization-induced reconstruction of the electronic structure near the Fermi level. However, the experimentally observed magnetic order is incommensurate and amplitude-modulated. The ambiguity in the powder neutron diffraction becomes more pronounced at lower temperatures, preventing an unambiguous determination of the magnetic structure and symmetry from powder diffraction alone. Therefore, the present results are insufficient to assign MnSb_2_ to an altermagnetic class or establish symmetry-protected momentum-dependent spin splitting. Further single-crystal neutron diffraction and spin-resolved spectroscopic measurements will be required to address this possibility. Because the experimental *q* vector is incommensurate and temperature-dependent, its exact representation in the DFT calculations would require a large commensurate approximant with substantial computational cost. The simplified collinear AFM model is therefore used only for qualitative analysis of magnetic stabilization and the electronic structure near the Fermi level.

**Figure 5:**
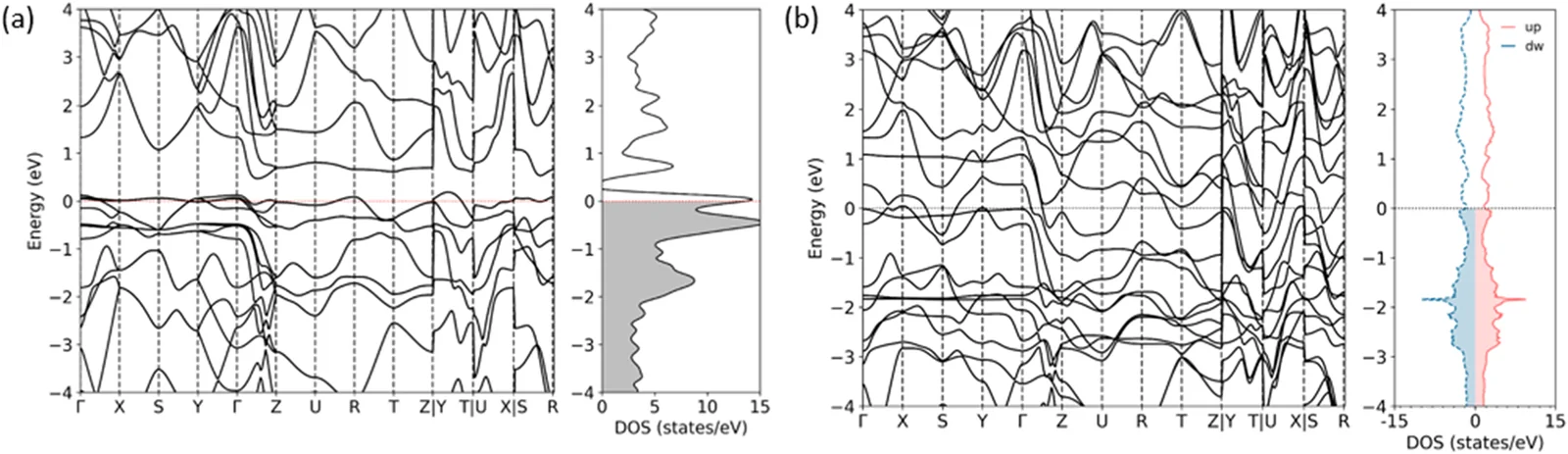
Calculated electronic band structure of MnSb_2_. (a) Nonmagnetic (spin-unpolarized) state. (b) Antiferromagnetic state.

## Conclusion

In summary, altermagnets have recently emerged as a distinct class of magnetic materials that combine collinear antiferromagnetic order with spin-split electronic band structures and zero net magnetization, offering new opportunities for quantum and spintronic functionalities. In this work, we establish MnSb_2_ as a chemically clean, pressure-stabilized marcasite-type compound hosting an unconventional magnetic ground state. The quenched MnSb_2_ phase remains stable at ambient pressure up to approximately 450 K, providing a sufficient thermal stability window for low-temperature measurements. Using high-pressure synthesis, thermodynamic measurements, neutron diffraction, and first-principles calculations, we show that MnSb_2_ does not develop a single, rigid magnetic order below a well-defined *T*_N_. Instead, magnetic entropy begins to be released near ∼230 K, accompanied by the appearance of incommensurate magnetic Bragg reflections below ∼200 K. A second thermodynamic anomaly near ∼118 K coincides with the emergence of additional magnetic peaks, indicating a reconstruction of the ordered state. Remarkably, specific heat and neutron refinements reveal that the magnetic structure continues to evolve throughout the ordered regime: the propagation vector shifts systematically with temperature (with the *b*-component trending toward 0.5). However, the available powder-diffraction data do not uniquely determine the detailed moment trajectory, rotation plane, or relative phase relationships among the magnetic components, and single-crystal neutron diffraction, including polarization analysis, will be required for an unambiguous determination. These findings establish MnSb_2_ as a rare stoichiometric platform in which an amplitude-modulated incommensurate structure with an SDW component remains highly tunable at low temperature, highlighting an unusual interplay between anisotropy, competing exchange interactions, and the underlying electronic structure.

## Supplementary Material



## Notes

Crystal structure and refinement of MnSb_2_; Atomic coordinates of MnSb_2_; calculated the total energy of different magnetic models; powder XRD results from the different batches; *M*(*H*) data of single crystals and polycrystals at 300 K; heat capacity and entropy analysis and *M*(*T*) measurements; PXRD results for the same batches; NMR spectrum of polycrystalline samples at 4.2K; Rietveld refinement of the 300 K powder neutron diffraction data for MnSb_2_; the single crystal X-ray diffraction sample picture and the synthesis design of MnSb_2_; refined atomic coordinates of MnSb_2_ using neutron diffraction at 300 K; basis vectors for the space group *Pnnm* at 300 K; basis vectors for the space group *P*1̅ at 200 K (PDF)
