# Supplementary material for: Pressure-Stabilized MnSb2 with Complex Incommensurate Magnetic Order
Source: J Phys Chem C Nanomater Interfaces. 2026 Sep 1;130(37):13036–45. doi: 10.1021/acs.jpcc.6c03519 (PMC13592071; doi:10.1021/acs.jpcc.6c03519)
Supplement: Supplementary file 1 [file jp6c03519_si_001.pdf]

Support Information

Pressure-Stabilized $MnSb_2$ with Complex Incommensurate Magnetic Order

Mingyu Xu[1,2,3], Matt Boswell[1,4], Qing-Ping Ding[2], Cheng Peng[1], Aashish Sapkota[2,3], Qiang Zhang[4], Danielle Yahne[4], Sergey. L. Bud'ko[2, 3], Yuji Furukawa[2,3], Paul. C. Canfield[2,3], Raquel A. Ribeiro[2,3], Weiwei Xie[1*]

[1]Department of Chemistry, Michigan State University, East Lansing, Michigan 48824, USA
[2]Ames National Laboratory, Iowa State University, Ames, Iowa 50011, USA
[3]Department of Physics and Astronomy, Iowa State University, Ames, Iowa 50011, USA
[4]Neutron Scattering Division, Oak Ridge National Laboratory, Oak Ridge, Tennessee 37831, USA

Corresponding Authors: Weiwei Xie (xieweiwe@msu.edu)

Table of Contents

Table S1 The crystal structure and refinement of $MnSb_2$ ……………………….........…………..S2
Table S2 Atomic coordinates of $MnSb_2$ ……………………………….....…….........…………..S2
Table S3 Calculated total energy of different magnetic models……...……………………..…..…..S2
Fig. S1. Powder XRD results from the different batches………………………..........…………..S3
Fig. S2. *M*(*H*) data of single crystals and polycrystals at 300K………………..........……………S4
Fig. S3. Heat capacity and entropy analysis and *M*(*T*) measurements ……...........……………S5
Fig. S4. PXRD results for the same batches……………………...……………............……………S6
Fig. S5. NMR spectrum of polycrystalline samples at 4.2K…...…………….........………………S7
Fig. S6. Rietveld refinement of the 300 K powder neutron diffraction data for $MnSb_2$…………...S9
Table S4 Refined atomic coordinates of $MnSb_2$ using neutron diffraction at 300 K…...………...S10
Table S5 Basis vectors for the space group *Pnnm* at 300 K…………………...........…....……..S10
Table S6 Basis vectors at 4 K…………………………..……………………..………………..S11
Table S7 Refined magnetic propagation vectors.…………………………………..……….…..S11
Table S8 Basis vector combinations.………………...………………………………..…….…..S11
Fig. S7. The synthesis design of $MnSb_2$………………………………………………………..S13

Table S1. The crystal structure and refinement of $MnSb_2$ at room temperature. Values in parentheses are estimated standard deviation from refinement.

**Chemical Formula**	**$MnSb_2$**
Formula Weight	298.44 g/mol
Space Group	*Pnnm*
	a = 6.0227(3) Å
lUnit Cell dimensions	b = 6.8893(3) Å
	c = 3.3240(2) Å
Volume	137.918(12) $Å^3$
Density (calculated)	7.186 g/cm^3
Absorption coefficient	23.579 mm^{-1}
F (000)	254
2θ range	9.00 to 80.26°
Reflections collected	3540
Independent reflections	478 [R_{int} = 0.0561]
Refinement method	Full-matrix least-squares on F^2
Data/restraints/parameters	478/0/11
Final R indices	R_1 (I>2σ(I)) = 0.0282; wR_2 (I > 2 σ(I)) = 0.0795
	R_1 (all) = 0.0289; wR_2 (all) = 0.0799
Largest diff. peak and hole	+2.072 $e^-/Å^3$ and -1.637 $e^-/Å^3$
R. M. S. deviation from mean	0.428 $e^-/Å^3$
Goodness-of-fit on F^2	1.229

Table S2. Atomic coordinates and equivalent isotropic atomic displacement parameters ($Å^2$) $MnSb_2$. (U_{eq} is defined as one-third of the trace of the orthogonalized U_{ij} tensor.) Values in parentheses are estimated standard deviations from refinement.

**Atoms**	**Wyck.**	***x***	***y***	***z***	**Occ.**	**U_{eq}**
**Sb**	4*g*	0.17921(5)	0.36252(4)	0	1	0.01228(11)
**Mn**	2*a*	0	0	0	1	0.01182(17)

Table S3. Calculated the total energy of different magnetic models.

	**NM**	**FM**	**AFM**
Magnetic Model 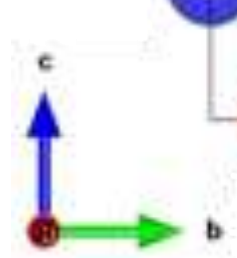	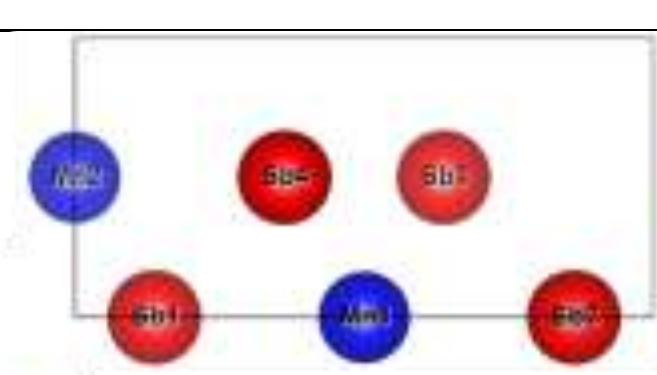	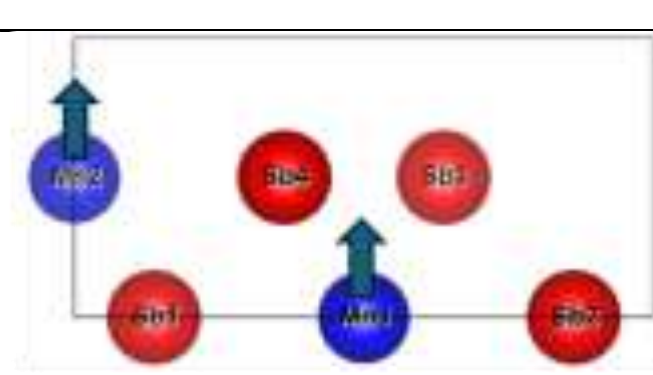	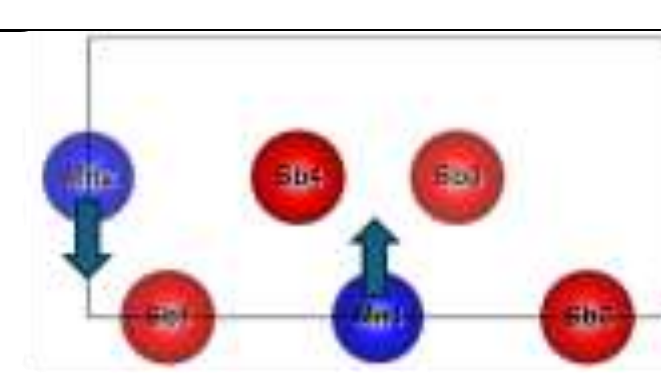
Total Energy	0 meV	-69.68 meV	-80.79 meV

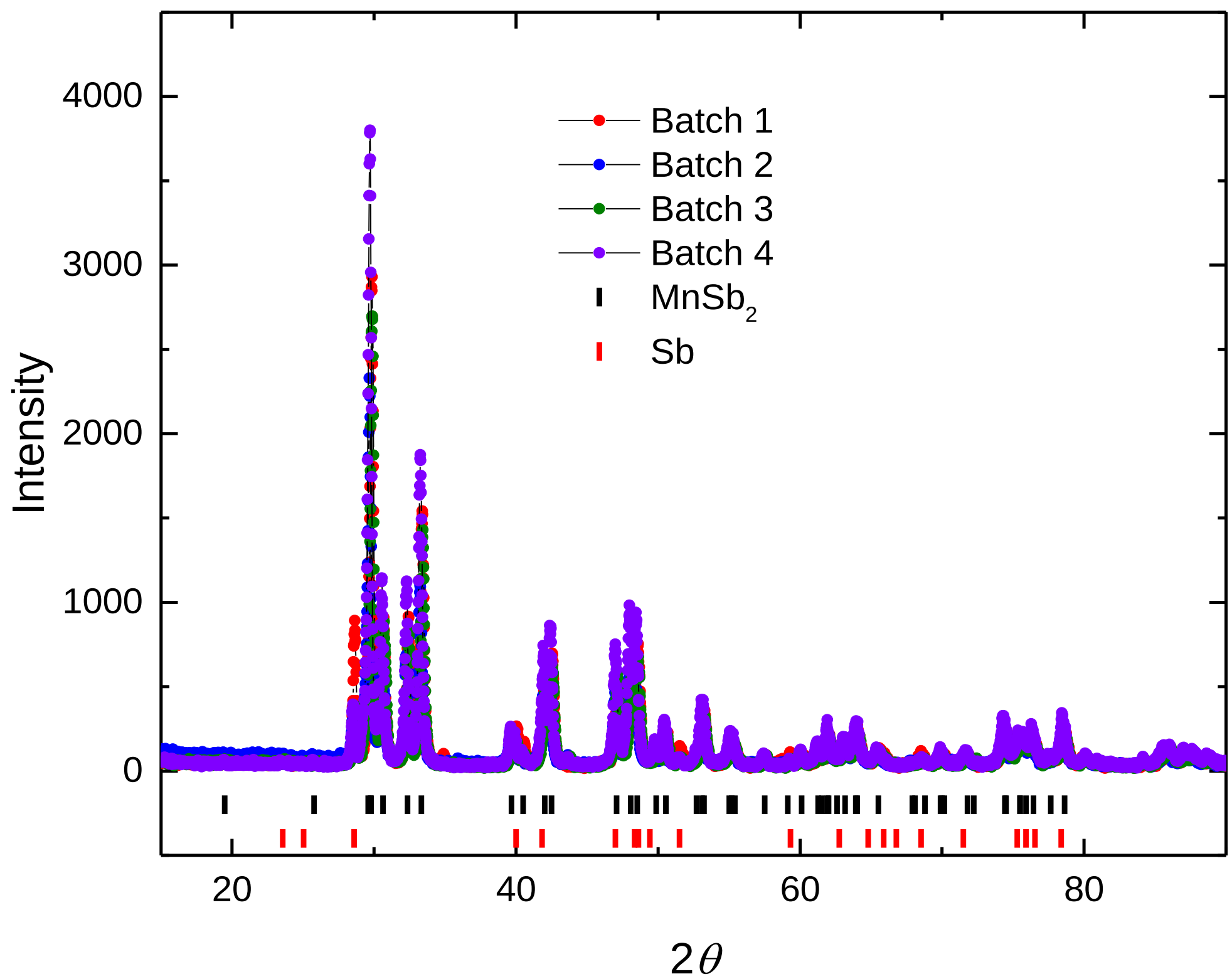

Figure S1, Powder XRD results from the different batches. The sample for XRD is a part of the chunk directly from synthesis without the separation of single crystals.

Figure S1 shows the powder XRD results from the different batches. The sample for XRD is a part of the chunk directly from synthesis without the separation of single crystals. To complete the reaction of Mn, a small excess of Sb (~1 wt%) was added.

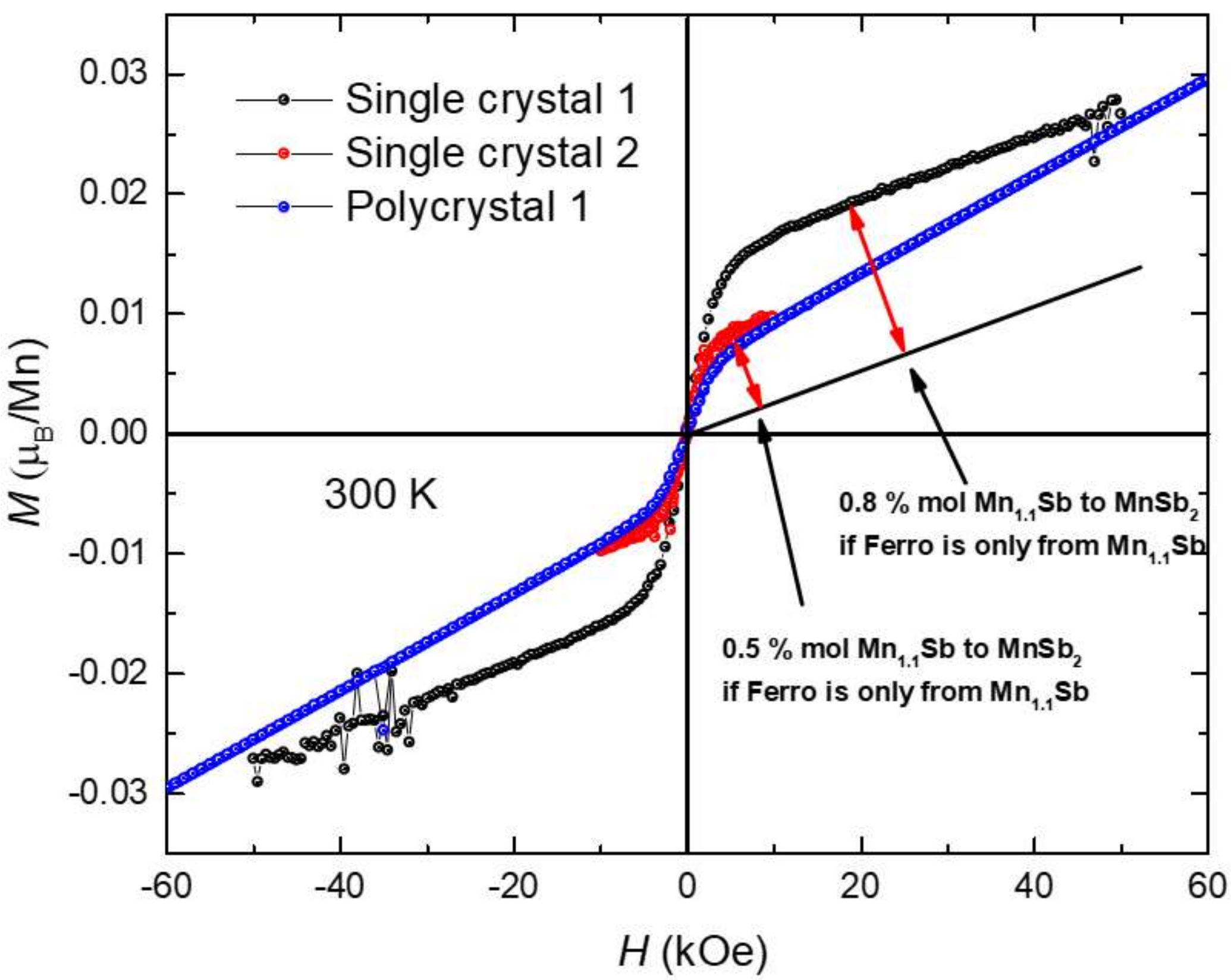

Figure S2, M(H) data of single crystals and polycrystals at 300 K. The mole ratio of $Mn_{1.1}Sb$ is estimated by assuming all the ferromagnetic single crystals come from $Mn_{1.1}Sb$.

Figure S2 shows *M* (*H*) data of single crystals and polycrystals at 300 K. The mole ratio of Mn1.1Sb is estimated by assuming all the ferromagnetic single crystals come from MnSb.

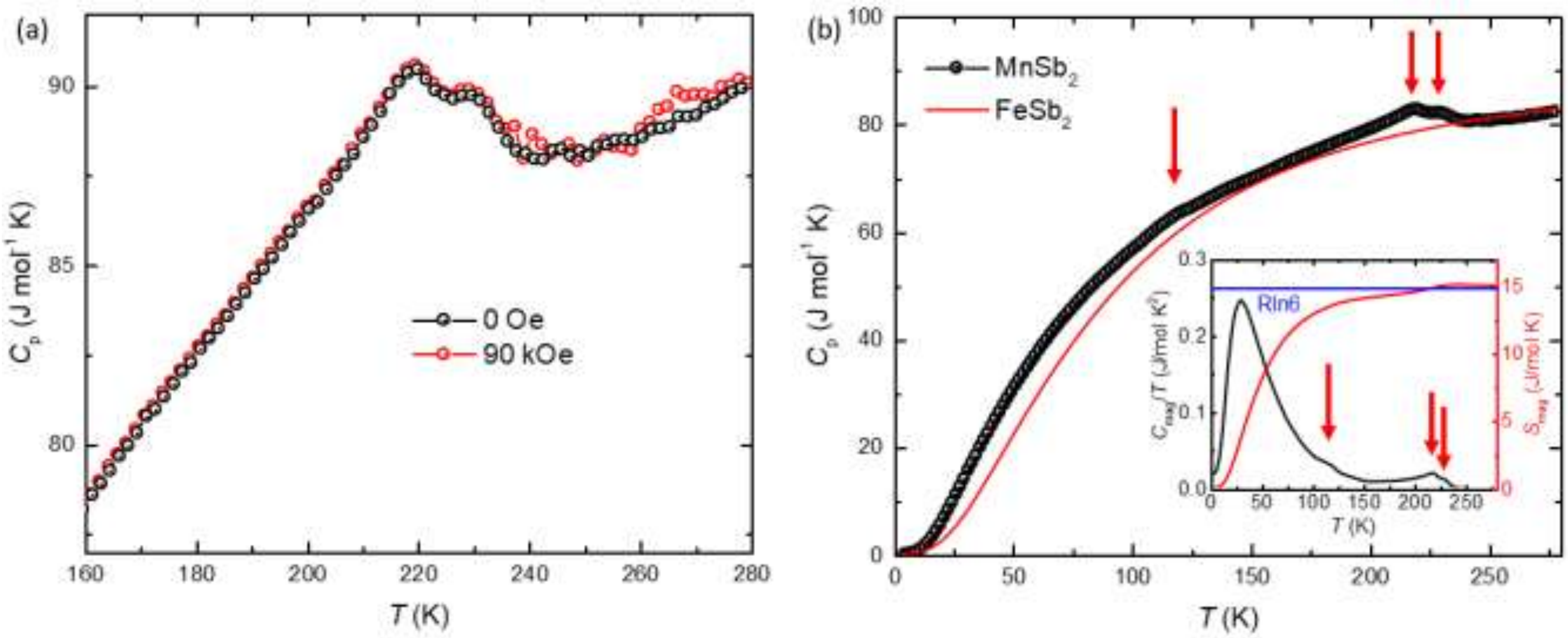

Figure S3, Temperature-dependent specific heat was measured in the temperature range from 150 K to 280 K and 2 K to 200 K. The measurement is taken on the N-grease with addenda taken with the same data points as the sample measurements in the temperature range from 150 K to 280 K. There is no clear difference between the field at 0 Oe or 90 kOe. Magnetic entropy is calculated with reference to $FeSb_2$ specific heat measurement.

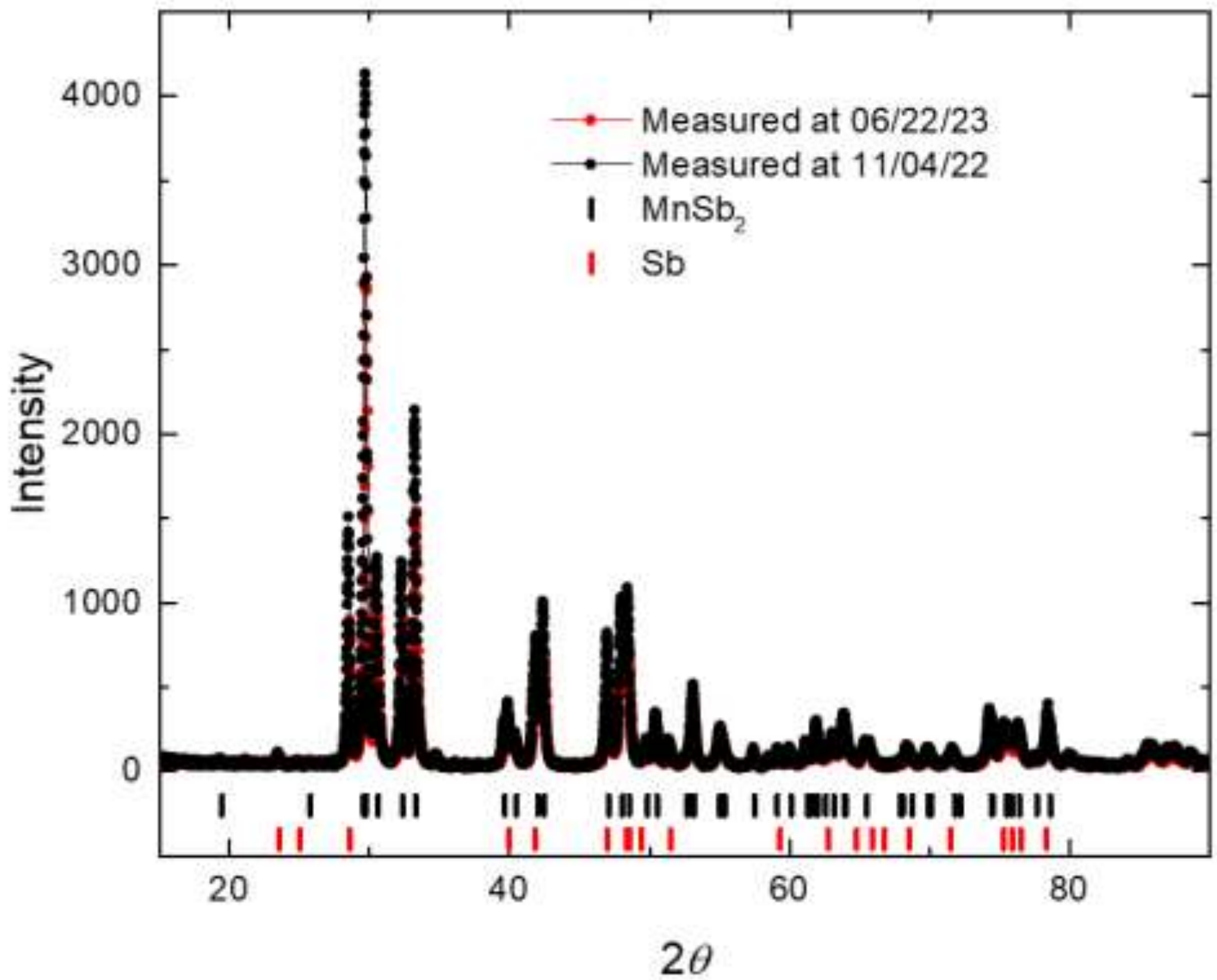

Figure S4. Powder XRD results from the same batch. The interval of the time is around six months. The highest intensity peak normalizes the intensity.

To assess the ambient stability of $MnSb_2$ over time, **Figure S4** shows the normalized powder X-ray diffraction (XRD) patterns of the same sample batch measured immediately after synthesis (black) and after six months of air exposure at room temperature (red). Despite $MnSb_2$ being a metastable phase at ambient pressure, no discernible changes in the diffraction pattern are observed, indicating that the phase remains structurally stable under ambient conditions for 6 months. This result suggests that $MnSb_2$ is stable at or below room temperature, with no evidence of decomposition or transformation over six months.

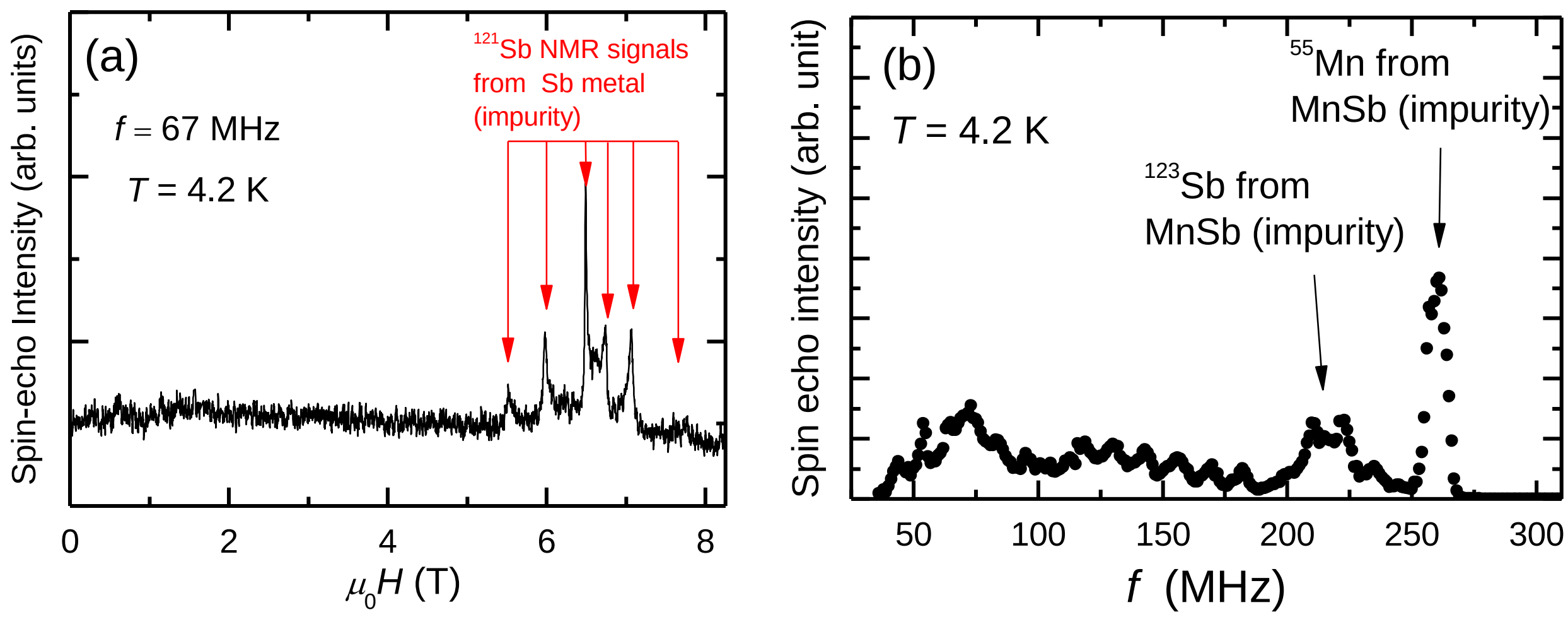

Figure S5. (a) NMR spectrum measured at T = 4.2 K by sweeping the magnetic field on polycrystalline powder samples. (b) Zero-field NMR spectrum at T = 4.2 K on polycrystalline powder samples.

We attempted to get information about the magnetic ground state of $MnSb_2$ from ^{55}Mn, ^{121}Sb, and ^{123}Sb NMR measurements at a low temperature of T = 4.2 K using the same sample from neutron diffraction measurements. **Figure S5*a*** shows the NMR spectrum, where a very broad spectrum from the highest magnetic field of our magnet (8.25 T) to zero field was observed. The relatively sharp lines detected from 5.5 T to 7.5 T (indicated by the red arrows) were assigned to a typical quadrupolar split ^{121}Sb NMR power lines from the impurity of Sb metal[1]. The intrinsic broad spectrum indicates that the hyperfine fields at the Mn and Sb sites are widely distributed in the magnetically ordered state, consistent with the inhomogeneous magnetic state as determined by the neutron diffraction measurements. Such an inhomogeneous magnetic state is also suggested by the very broad zero-field NMR spectrum shown in **Figure S5*b***. The signals around 250-260 MHz and 200-230 MHz are from ^{55}Mn and ^{123}Sb, respectively, in the impurity of the ferromagnetic $Mn_{1.1}Sb$[2]. Although the broad spectrum is not fully resolved at present, we consider that the signals below 200 MHz are due to ^{55}Mn and ^{121}Sb NMR signals in antiferromagnetically ordered $MnSb_2$. This again suggests the large distributions of hyperfine fields at the Mn and Sb sites, consistent with the NMR spectrum, indicating the inhomogeneous magnetic state.

To investigate the possibility that high-pressure synthesized $MnSb_2$ exhibits magnetic ordering above room temperature (T_n > 300 K), powder neutron diffraction measurements were conducted to detect either additional magnetic Bragg reflections or intensity enhancements of the

nuclear Bragg peaks consistent with the P*nmm* crystal symmetry. In general, antiferromagnetic (AFM) ordering gives rise to new magnetic reflections, while ferromagnetic ordering manifests as enhanced intensity at the positions of nuclear Bragg peaks. **Figure S6*a*** presents the neutron diffraction pattern collected at 300 K, along with the corresponding Rietveld refinement. All observed peaks are consistent with the P*nmm* symmetry of $MnSb_2$, and no additional magnetic peaks are detected. The refined lattice parameters from the neutron diffraction data are *a* = 6.0162(2) Å, *b* = 6.8824(3) Å, and *c* = 3.3226(1) Å, which are in good agreement with the values obtained from the X-ray diffraction refinement. Minor impurity phases were identified as MnSb (2.7 wt%), MnO (0.3 wt%), Sb (3.6 wt%), and boron nitride (BN, 0.07 wt%), which account for the weak reflections not indexed to the primary phase. Statistical parameters from the refinement, including all the phases in the model, are R_{wp} = 6.0% and GOF= 3.2, indicating a reasonable refinement. The site occupancy, atomic coordinates, and isotropic displacement parameters obtained from the refinement are summarized in **Table S4**.

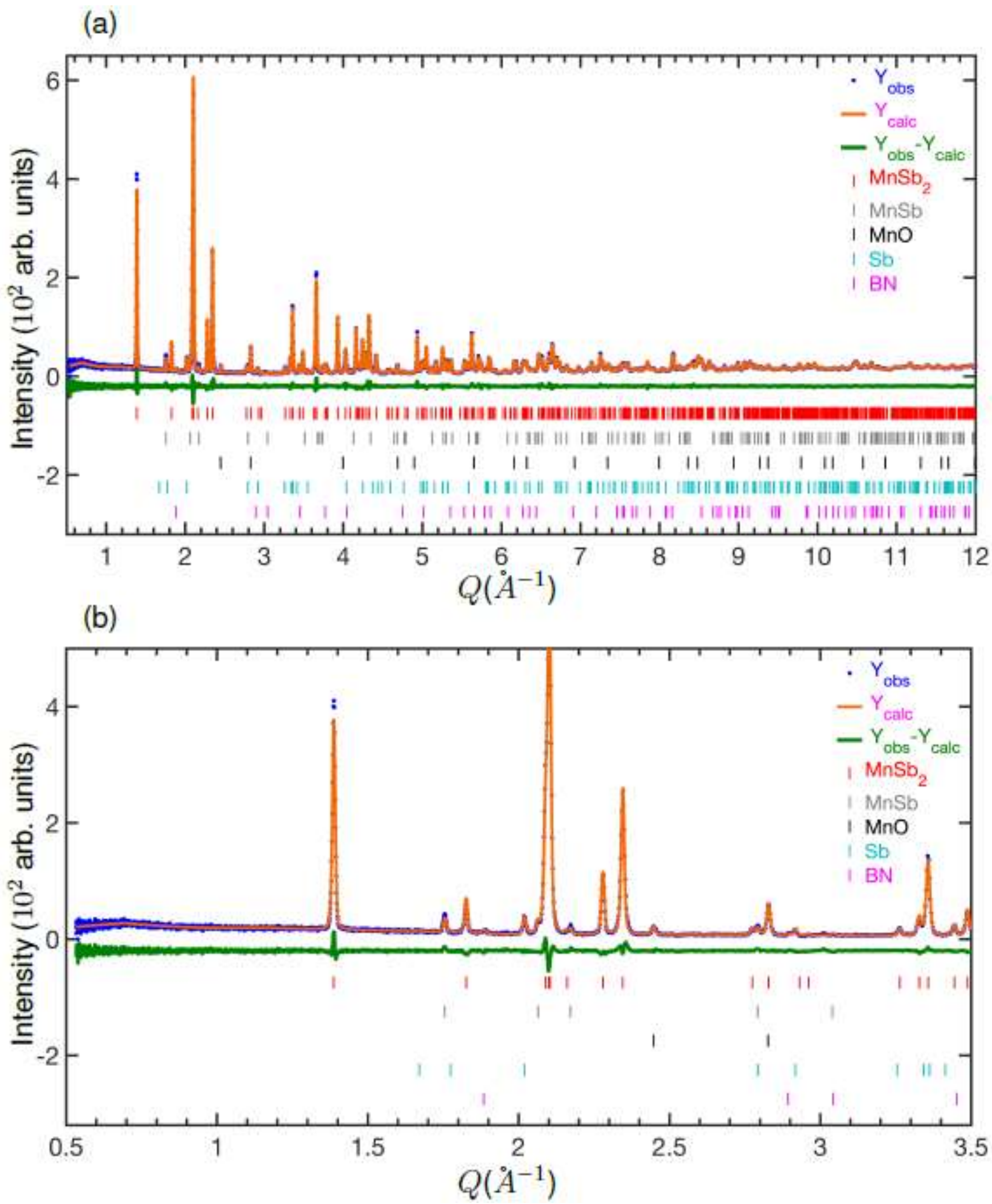

Figure S6. (*a*) Rietveld refinement of the 300 K powder neutron diffraction data for $MnSb_2$, yielding a weighted profile R-factor (R_{wp}) of 6.0% and a goodness of fit (GOF) of 3.2. The blue dots represent the experimental data, while the orange line corresponds to the calculated pattern. The green line indicates the difference between the observed and calculated intensities. Vertical tick marks represent the allowed Bragg reflection positions for the identified phases: $MnSb_2$ (red), Sb (gray), MnSb (black), MnO (cyan), and boron nitride (BN, magenta). **(*b*)** Enlarged view of the low-Q region from panel **(*a*)**, emphasizing the absence of additional magnetic Bragg peaks, indicating no long-range magnetic ordering at room temperature.

Table S4. Refined atomic coordinates and the equivalent isotropic displacement parameters of $MnSb_2$ at 300 K.

**Atoms**	**Wyck.**	***x***	***y***	***z***	**Occ.**	U_{eq}
**Sb**	4*g*	0.1799(1)	0.3624(1)	0	1	0.0080(2)
**Mn**	2*a*	0	0	0	1	0.0060(3)

Table S5. Basis vectors for the space group *Pnnm* with k = (0, 0, 0). The decomposition of the magnetic representation for the Mn site (0, 0, 0) is $\Gamma_{Mag} = 1\Gamma_1^1 + 2\Gamma_3^1 + 2\Gamma_5^1 + 1\Gamma_7^1$. The atoms of the nonprimitive basis are defined according to 1: (0, 0, 0), 2: (1/2, 1/2, 1/2).

IR	BV	Atom	BV components					
			$m_{\parallel a}$	$m_{\parallel b}$	$m_{\parallel c}$	$im_{\parallel a}$	$im_{\parallel b}$	$im_{\parallel c}$
Γ_1	ψ_1	1	0	0	4	0	0	0
		2	0	0	-4	0	0	0
Γ_3	ψ_2	1	4	0	0	0	0	0
		2	4	0	0	0	0	0
	ψ_3	1	0	4	0	0	0	0
		2	0	-4	0	0	0	0
Γ_5	ψ_4	1	4	0	0	0	0	0
		2	-4	0	0	0	0	0
	ψ_5	1	0	4	0	0	0	0
		2	0	4	0	0	0	0
Γ_7	ψ_6	1	0	0	4	0	0	0
		2	0	0	4	0	0	0

Table S6. Symmetry-allowed magnetic basis vectors obtained from representation analysis for Pnnm space group with k-vectors (0 0.470 0.375) at 4 K.

IR	BV	Mn (0.5 0.5 0)	Mn (0 0 0.5)
Γ_1	ψ_1	(1 0 0)	(0.95+0.32*i* 0 0)
	ψ_2	(0 1 0)	(0 -0.95-0.32*i* 0)
	ψ_3	(0 0 1)	(0 0 – 0.95-0.32*i*)
Γ_2	ψ_4	(1 0 0)	(-0.95-0.32*i* 0 0)
	ψ_5	(0 1 0)	(0 0.95+0.32*i* 0)
	ψ_6	(0 0 1)	(0 0 0.95+0.32*i*)

Table S7. Refined magnetic propagation vectors, $q = (0, q_b, q_c)$, obtained from powder neutron diffraction at selected temperatures. The b-component systematically approaches 0.5 upon cooling, while the c-component remains nearly constant. Although the complete magnetic structure cannot be uniquely determined from the powder data, the propagation vectors are constrained by the magnetic Bragg peak positions.

	200 K	100 K	4 K
q	(0,0.3975,0.3783)	(0,0.4549,0.3759)	(0,0.4781,0.3759)

Table S8*a* Basis vector combinations at 200 K. 1 indicates that it is a real component and is active, while i indicates an active imaginary component.

Basis Vectors	R-factor	χ^2	Type
100011	19.03	3.42	Sinusoidal
i00011	19.19	3.42	Helical
000011	20.75	3.43	Sinusoidal
i00111	26.15	3.39	Helical
100111	26.22	3.39	Sinusoidal
iii011	26.90	3.40	Helical
000111	27.36	3.39	Sinusoidal
0001ii	27.45	3.39	Helical
11100	42.51	3.89	Sinusoidal
111i00	39.76	3.89	Helical
110001	34.42	3.61	Sinusoidal
011100	60.89	4.98	Sinusoidal

Table S8*b* Basis vector combinations at 4 K. 1 indicates that it is a real component and is active, while i indicates an active imaginary component.

Basis Vectors	R-factor	χ^2	Type
iii011	33.74	5.75	Helical
1ii01i	34.04	5.78	Helical
111000	37.01	5.92	Sinusoidal
101i1i	35.04	5.76	Helical

000111	40.53	6.00	Sinusoidal
100011	43.49	6.52	Sinusoidal
i00011	43.75	6.51	Helical
000011	47.22	6.71	Sinusoidal
i11111	34.61	5.73	Helical
111111	34.72	5.73	Sinusoidal
ii1000	36.13	5.87	Helical
000i1i	38.41	5.94	Helical
100i1i	37.06	5.83	Helical

To further probe potential magnetic contributions in $MnSb_2$, **Figure S6*b*** highlights the low-*Q* region of the neutron diffraction pattern, where magnetic scattering is typically enhanced due to the *Q*-dependence of the magnetic form factor. The absence of additional Bragg reflections in this region provides compelling evidence against the presence of long-range magnetic ordering at room temperature. In particular, the lack of magnetic peaks strongly suggests that antiferromagnetic (AFM) ordering with a non-zero propagation vector ($k \neq (0, 0, 0)$) is absent in this compound. Nonetheless, the possibility of ferromagnetic ordering corresponding to $k = (0, 0, 0)$ cannot be excluded solely based on the absence of additional reflections. In such a case, magnetic ordering would manifest as an enhancement of nuclear Bragg peak intensities. However, our Rietveld refinement shows no observable intensity increase at the nuclear peak positions, and no systematic deviation between observed and calculated intensities was detected. To definitively assess the presence of ferromagnetic ordering and to estimate an upper bound for the ordered Mn moment, magnetic structure refinements were performed using the FullProf suite with magnetic subgroups of the *Pnmm* space group. Symmetry-allowed magnetic configurations were derived from a group-subgroup analysis of the propagation vector $k = (0, 0, 0)$, implemented via SARAh-Representational Analysis. Four irreducible representations (IRs) and their corresponding basis vectors (BVs) were identified, as listed in **Table S5**. Among them, IRs Γ_3 (with BV ψ_2) and Γ_5 (with BV ψ_5), which describe ferromagnetic alignment of Mn moments within the *ab*-plane, produced the best magnetic refinements, with magnetic *R*-factors (R_{mag}) below 5. Despite these fits, the refined ordered moment was consistently small, with a total magnetic moment of μ_{tot} that should be much smaller than 0.2 μ_B/Mn, indicating the absence of significant static magnetic ordering in $MnSb_2$ at room temperature.

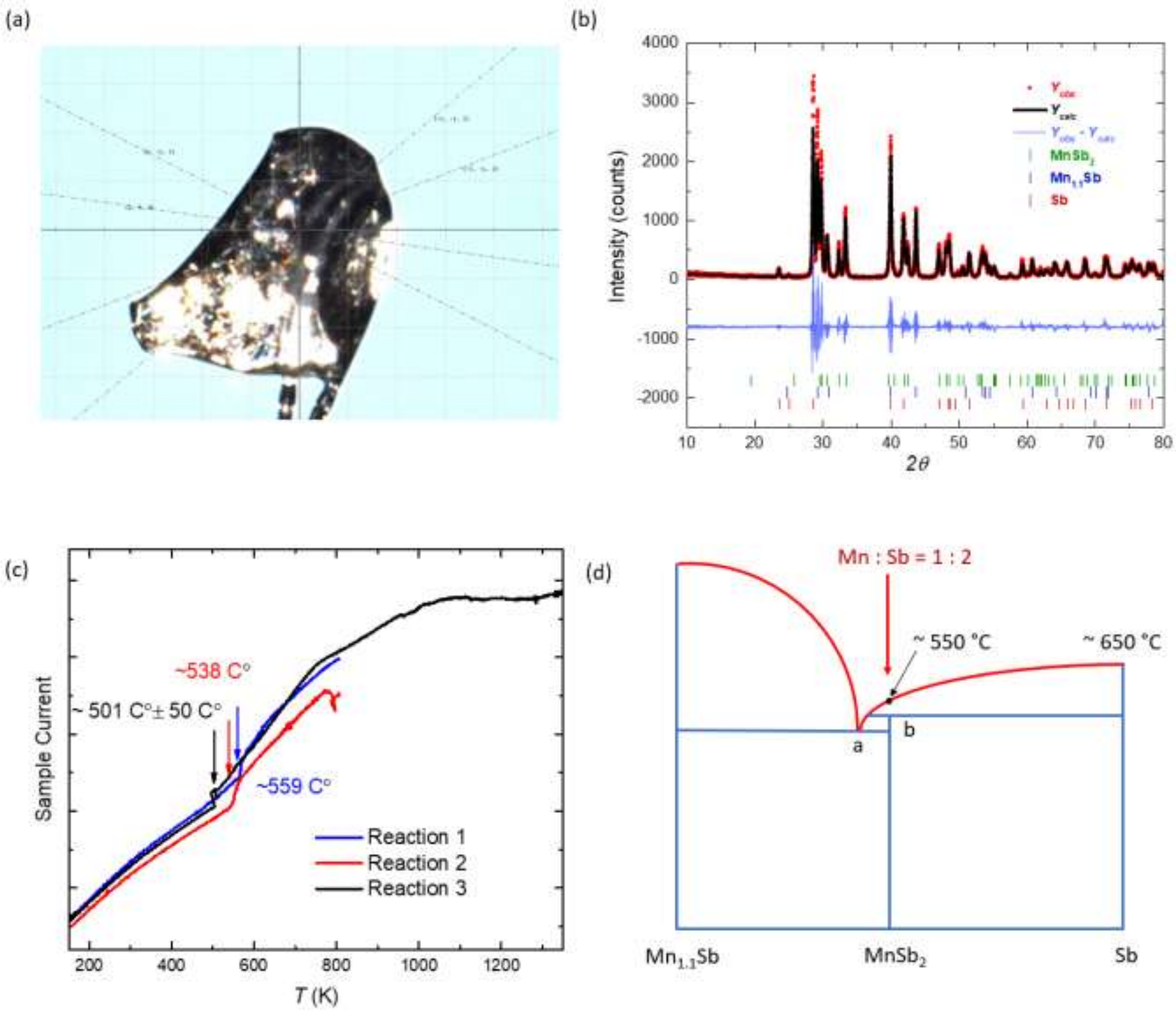

Figure S7. ***(a)*** The sample used to determine the crystallographic direction of $MnSb_2$ is shown. ***(b)*** The PXRD data are shown for solid-state reactions at temperatures above 700 °C. ***(c)*** The sample current as a function of temperature is shown for different $MnSb_2$ reactions. ***(d)*** The phase diagram is shown. A point is the eutectic point, and the b point is the peritectic point. The red curve indicates the liquidus lines.

Reference

(1) Hewitt, R. R.; Williams, B. F. Nuclear Quadrupole Interaction of Sb121 and Sb123 in Antimony Metal. Phys Rev 1963, 129, 1188. https://doi.org/10.1103/PhysRev.129.1188

(2) Nagarajan, V.; Vijayaraghavan. Nuclear Magnetic Resonance in Ferromagnetic MnSb and $Cr_xMn_{1-x}Sb$. J Phys Soc Jpn 1972, 33, 88-91. https://doi.org/10.1143/JPSJ.33.88
